# Enhanced Handoff Scheme for Downlink-Uplink Asymmetric Channels in Cellular Systems

**DOI:** 10.1155/2013/241483

**Published:** 2013-12-31

**Authors:** Sunghyun Cho, Ji-Su Kim, Jae-Hyun Kim

**Affiliations:** ^1^Department of Computer Science and Engineering, Hanyang University, 55 Daehak-ro, Sangrok-gu, Ansan, Gyeonggi-do 426-791, Republic of Korea; ^2^Department of Electrical and Computer Engineering, Ajou University, San 5 Woncheon-dong, Yeongtong-gu, Suwon, Gyeonggi-do 443-749, Republic of Korea

## Abstract

In the latest cellular networks, data services like SNS and UCC can create asymmetric packet generation rates over the downlink and uplink channels. This asymmetry can lead to a downlink-uplink asymmetric channel condition being experienced by cell edge users. This paper proposes a handoff scheme to cope effectively with downlink-uplink asymmetric channels. The proposed handoff scheme exploits the uplink channel quality as well as the downlink channel quality to determine the appropriate timing and direction of handoff. We first introduce downlink and uplink channel models that consider the intercell interference, to verify the downlink-uplink channel asymmetry. Based on these results, we propose an enhanced handoff scheme that exploits both the uplink and downlink channel qualities to reduce the handoff-call dropping probability and the service interruption time. The simulation results show that the proposed handoff scheme reduces the handoff-call dropping probability about 30% and increases the satisfaction of the service interruption time requirement about 7% under high-offered load, compared to conventional mobile-assisted handoff. Especially, the proposed handoff scheme is more efficient when the uplink QoS requirement is much stricter than the downlink QoS requirement or uplink channel quality is worse than downlink channel quality.

## 1. Introduction

In cellular systems, the downlink and uplink channel qualities for a mobile station (MS) can be different for various reasons. In frequency division duplex (FDD) systems, downlink-uplink channel asymmetry may occur because different frequency bands are allocated to the downlink and uplink channels. Although the same frequency band is used for the downlink and uplink channels in time division duplex (TDD) systems, channel asymmetry can still occur if the downlink and uplink have different interference models. Moreover, unlike traditional voice services, the latest data services, like social network service (SNS), user-created contents (UCC), and wireless peer-to-peer (P2P), can have different service requirements for downlink and uplink channels. For example, streaming service requires a high data rate only in the downlink channel, whereas a data uploading service, for example, sending an e-mail with large attached files, requires a high data rate only in the uplink channel. This asymmetry of service requirements and traffic patterns can lead to a downlink-uplink asymmetric channel condition and affect handoff performances. Therefore, to reduce the call dropping rate and degradation of the service quality in the handoff region, it is more effective for the downlink-uplink asymmetric services to consider both the downlink and the uplink signals in a handoff decision.

A considerable amount of research on handoffs has been published over the past few decades [1–14]. The conventional handoff schemes can be categorized into mobile-assisted handoff (MAHO), mobile-controlled handoff (MCHO), and network-controlled handoff (NCHO) according to where the handoff decision is performed. In addition, the handoff schemes can be classified into hard handoffs and soft handoffs according to the continuity of the connection during the handoff process.

In conventional CDMA systems, the most commonly used scheme has been mobile-assisted soft handoff. The mobile-assisted handoff algorithm has been presented in [1–7]. In MAHO, a serving base station (BS) broadcasts its set of neighbor BSs. An MS periodically measures the pilot signal strength of all neighbor BSs in the set and reports a measurement result to a serving BS. The serving BS determines handoff execution and selects a target BS based on the measurement result. During a hard handoff process [2–5], the data channel is temporarily disconnected, creating a service interruption. However, with a soft handoff algorithm [6, 7], the data channel is maintained without a service interruption. In a soft handoff, an active BSs set, which consists of BSs with signal strengths above a predetermined threshold, is defined and maintained. An MS periodically updates the active BSs set based on the received signal strengths. It can receive the downlink data from multiple BSs in an active BSs set, simultaneously, using an RAKE receiver [6, 7]. This ability prevents service interruption in the handoff region. However, the handoff algorithms in CDMA systems usually consider only the downlink signal quality in determining handoff executions and directions.

In contrast, the handoff schemes in GSM systems consider uplink as well as downlink signal quality [1, 8–10]. An MS measures the quality of the downlink signals received from a serving BS and the neighbor BSs every 0.5 sec. The serving BS measures the uplink signal quality characteristics, such as signal strength, BER, and the distance from the BS, based on the adaptive timing advance parameter. However, in GSM systems, downlink signal quality is a dominant criterion in determining a handoff direction, although the serving BS measures the uplink signal quality [1, 10].

Recently, handoff schemes for OFDM systems have been presented in the research papers and the IEEE 802.16e/m standard specification [11–14]. In IEEE 802.16m, three different types of handoff algorithms are defined: mobile-assisted handoff (MAHO), macrodiversity handoff (MDHO), and fast base-station switching (FBSS). MAHO in IEEE 802.16m is almost the same as with MAHO in CDMA systems, but with a default operation mode. MDHO and FBSS are optional handoff modes, defined to support a make-before-break handoff. It is necessary to maintain a diversity set and an anchor BS in these handoff schemes. A diversity set is a list of active base stations, and an anchor BS is a node where mobile stations are registered and synchronized. The basic idea of MDHO is the same as for soft handoff in CDMA systems, but the method to receive downlink data from multiple BSs is different. In MDHO, an MS dynamically updates a diversity set based on the mean carrier-to-interference-and-noise ratio (CINR) from the neighbor BSs. In contrast, an MS communicates only with the anchor BS in FBSS. However, FBSS significantly reduces handoff latency by removing the network reentry process from handoff procedures.

Although previous works have provided a variety of handoff algorithms focusing on the reduction of handoff latency or service interruption time, they have not thoroughly tackled the effects of channel asymmetry and service requirement asymmetry between downlinks and uplinks on handoff algorithm design and handoff performance. Therefore, we mathematically model downlink and uplink OFDM channels to investigate the effects of channel asymmetry on the handoff algorithm and handoff performance. Based on these channel models, we propose a handoff scheme that considers the downlink and uplink channel qualities in the handoff decision algorithm to reduce the handoff-call dropping probability and service interruption time. The rest of this paper is organized as follows.  Section 2 presents downlink and uplink channel models that takes inter-cell interference into consideration.  Section 3 describes the proposed handoff schemes. The performance evaluation results are given in Section 4. Finally, we present the study conclusions in Section 5.

## 2. System and Channel Models

In this section, we derive the downlink and uplink average SINR models by geometric analysis and describe the channel asymmetric cases based on the derived SINR models.

### 2.1. System Model

**Figure 1:**
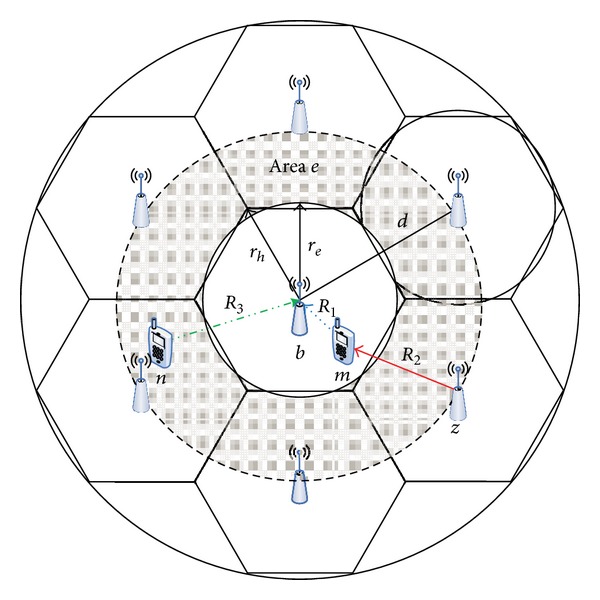
Cell deployment.

This paper considers TDD-based OFDMA systems in which downlink and uplink channels are time-division multiplexed.  Figure 1 depicts the considered cell structure. The frequency reuse factor is assumed to be one, and consequently every adjacent cell can cause inter-cell interference with the center cell. All BSs are assumed to be time synchronized, but BSs are not coordinated in the resource allocation. So each BS cannot sense the traffic of the neighbor cells. In addition, MSs are assumed to be uniformly distributed in a cell. To simplify the analysis of the channel modeling, we consider only path loss and shadowing. Fast fading is not considered because its effect is lessened in the time-averaged channel quality that is usually exploited as a handoff decision criterion. Under these assumptions, we derive downlink-uplink channel model between BS *b* and MS *m* located on the center cell, as shown in Figure 1. Received signal strength (RSS) is a typical channel quality metric, but other metrics, such as bit error rate (BER) or signal-to-interference-and-noise ratio (SINR), can be used as well. In the proposed channel model, SINR is chosen as a channel quality metric to reflect the intercell interference. To derive the SINR model, various variables are introduced, as shown in Table 1.

We first derive a path loss model based on [15]. As shown in Figure 1, three different distances are considered in deriving a path loss model. *R*_1_ is the distance from a serving BS to a target MS, and it is used for the downlink and an uplink signal path loss. *R*_2_ is the distance from the target MS to a neighbor BS, and it is used for downlink interference path loss. *R*_3_ is the distance from a serving BS to an MS on a neighbor cell, and it is used for uplink interference path loss. The probability density functions for *R*_1_, *R*_2_, and *R*_3_ are derived as

(1) $$\begin{array}{c} f_{R_{1}}\left(r\right)=\frac{2r}{r_{e}^{2}}, \end{array}$$

(2) $$\begin{array}{c} f_{R_{2}}\left(r\right)=\frac{\sqrt{4d^{2}r_{e}^{2}-{\left(d^{2}+r_{e}^{2}-r^{2}\right)}^{2}}}{\pi r_{e}^{2}d^{2}}, \end{array}$$

(3) $$\begin{array}{c} f_{R_{3}}\left(r\right)≃\frac{2r}{{\left(r_{e}+d\right)}^{2}-{\left(d/2\right)}^{2}}. \end{array}$$

To calculate the average path loss, we derive the *γ*th moment of each *R*_1_, *R*_2_, and *R*_3_. From (1), the *γ*th moment of distance *R*_1_ between the serving BS *b* and the MS *m* is derived as

(4) $$\begin{array}{c} E\left[r_{b\to m}^{\gamma }\right]=E\left[r_{m\to b}^{\gamma }\right]=\overset{r_{e}}{\underset{ɛ}{\int }}\frac{2r^{\gamma +1}}{r_{e}^{2}}dr. \end{array}$$

If the distance between the BS and the MS is smaller than *ɛ*, then the distance is regarded as *ɛ*. The *γ*th moment of distance *R*_2_ from the neighbor BS *z* to the MS *m* is derived as

(5) $$\begin{array}{c} E\left[r_{z\to m}^{\gamma }\right]=\overset{d+r_{e}}{\underset{d-r_{e}}{\int }}\frac{r^{\gamma }}{\pi \cdot r_{e}^{2}}\cdot \frac{\sqrt{4d^{2}r_{e}^{2}-{\left(d^{2}+r_{e}^{2}-r^{2}\right)}^{2}}}{d^{2}}dr, \end{array}$$

where $r_{e}≃r_{h}=d/\sqrt{3}$. From (3), the *γ*th moment of distance *R*_3_ from the MS *n* in a neighbor cell to the serving BS *b* is derived as

(6) $$\begin{array}{c} E\left[r_{n\to b}^{\gamma }\right]=\overset{d+r_{e}}{\underset{d/2}{\int }}\frac{2r^{\gamma +1}}{{\left(r_{e}+d\right)}^{2}-{\left(d/2\right)}^{2}}dr. \end{array}$$

Second, shadowing is modeled on a log-normal distribution with zero mean. The expectation of a log normally distributed random variable with a mean of *m*_*ζ*_ = 0 and a variance of *σ*_*ζ*_^2^ is derived as

(7) E[10−ζ/10]  =10−mζ/10·exp⁡⁡{((ln⁡10/10)σζ)22}  =exp⁡⁡{((ln⁡10/10)σζ)22}.

### 2.2. Downlink Channel Model

The expected received signal power at the desired MS *m* from the serving BS *b* is written as

(8) E[Pb→m(r)]=Pmax⁡,BS(t)·α¯·E[rb→mγ]·E[10−ζb→m/10]=Pmax⁡,BS(t)·α¯·∫ɛre2rγ+1re2dr ·exp⁡⁡{((ln⁡10/10)σζb→m)22}.

In OFDMA systems, users can share tones that form a frame. Subsets of tones are assigned to each user based on a resource allocation algorithm. The downlink resource allocation ratio, $\bar{\alpha }$, can be different for each user and it affects the received power of a downlink channel. The expectation of the received interference power from neighbor BSs to the desired MS *m* is written as

(9) E[Im(r)]=θ360°  ·∑z≠b{∑n∈z(Pmax⁡,BS(t)·E[αn])·E[rz→mγ]·E[10−ζz→m/10]}≃Pmax⁡,BS(t)·6·δ¯·α¯2·θ360°    ·∫d−red+rerγπ·re2‍  ·4d2re2−(d2+re2−r2)2d2dr·exp⁡⁡{((ln⁡10/10)σζz→m)22}.

MSs in neighbor cells can use the same carrier frequency as the MS *m*. Thus, the first $\bar{\alpha }$ of the product ${\bar{\alpha }}^{2}$ represents the received interference power portion of the maximum transmitting power of the BS, and the second $\bar{\alpha }$ represents the probability of receiving interference from neighbor cells. The more the serving cell and the neighbor cells share the same tones, the higher $\bar{\alpha }$ becomes. Thus, the probability of receiving interference is proportional to $\bar{\alpha }$. Using (8) and (9), the downlink signal-to-noise-interference ratio (SINR) at the desired MS *m* is derived as

(10) $$\begin{array}{c} \Gamma_{b\to m}^{\left(r\right)}=\frac{E\left[P_{b\to m}^{\left(r\right)}\right]}{\left(E\left[I_{m}^{\left(r\right)}\right]+N_{0}\right)}. \end{array}$$

### 2.3. Uplink Channel Model

The expected received signal power from the desired MS *m* to the serving BS *b* is expressed as

(11) E[Pm→b(r)]=Pmax⁡,MSserving(t)·β¯·E[rm→bγ]·E[10−ζm→b/10]=Pmax⁡,MSserving(t)·β¯·∫ɛre2rγ+1re2dr‍·exp⁡⁡{((ln⁡10/10)σζm→b)22}.

When the distance between two adjacent BSs, *d*, is larger than 2*r*_*e*_, adjacent cells can be overlapped with each other. MSs in the overlapped area are assumed to be connected to the nearest BS and cause the uplink receive interference from neighbor cells. The uplink resource allocation ratio per MS, $\bar{\beta }$, affects the received signal and interference power as $\bar{\alpha }$. The expected received interference power from MSs in neighbor cells received by the serving BS *b* is written as

(12) E[Ib(r)]=θ360°·∑nPmax⁡,MS(t)neighbor·E[βn]·E[rn→bγ] ·E[10−ζn→b/10]≃{Pmax⁡,MSneighbor(t)·6·δ¯·β¯2·θ360°   ·∫d/2d+re2rγ+1(re+d)2−(d/2)2dr ·exp⁡{((ln⁡10/10)σζn→b)22},(d≤2re)Pmax⁡,MSneighbor(t)·6·δ¯·β¯2·θ360°  ·∫red+re2rγ+1(re+d)2−re2dr ·exp⁡⁡{((ln⁡10/10)σζn→b)22},(d>2re).

From (11) and (12), the uplink SINR from the desired MS to the serving BS *b* is derived as

(13) $$\begin{array}{c} \Gamma_{m\to b}^{\left(r\right)}=\frac{E\left[P_{m\to b}^{\left(r\right)}\right]}{\left(E\left[I_{b}^{\left(r\right)}\right]+N_{0}\right)}. \end{array}$$

### 2.4. Channel Asymmetric Cases

In this section, we introduce a few numerical results to demonstrate asymmetric channel conditions in cellular systems. We focus on the notable asymmetric cases based on the derived SINR model. As specified in (10) and (13), various parameters such as *α*, *β*, *r*_*e*_, *d*, *σ*_*ζ*_, *γ*, *N*_0_, *P*_max⁡,BS_^(*t*)^, and *P*_max⁡,MS_^(*t*)^ affect the downlink and uplink SINRs. The values of some parameters are determined after a system is deployed, and they are not frequently changed. For example, the path loss exponent factor *γ*, the noise distribution factor *N*_0_, and the shadowing distribution factor *σ*_*ζ*_ can be regarded as having fixed values when we assume that every wireless path has the same environment. These fixed parameters do not affect the channel symmetry. However, some parameters, such as *α*, *β*, and *d*, can have variable values during communications. The downlink and uplink resource allocation ratios per user, *α* and *β*, are instantaneously varied according to the service type and the statistical distribution of MSs. The distance between two adjacent BSs, *d*, can also be varied according to the change of cell planning or the deployment of a femto-cell BS. These variable parameters can cause an asymmetric channel to form [16]. For these reasons, we investigate downlink and uplink asymmetric cases according to variable parameters, such as the distance *d* between two BSs and the downlink and uplink resource allocation ratios, *α* and *β*.

**Figure 2:**
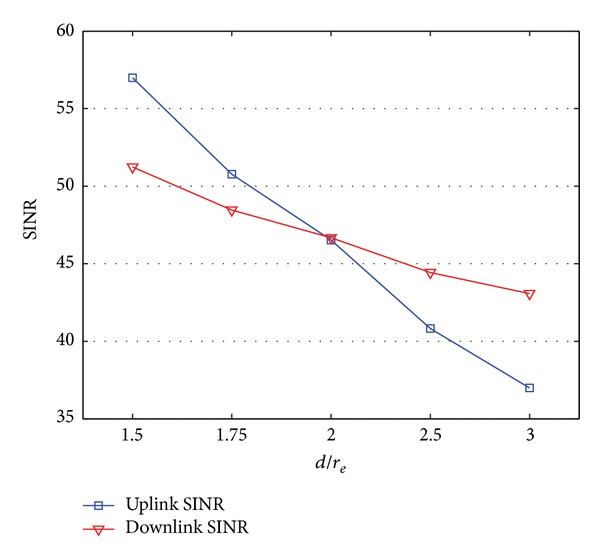
SINR according to neighbor cell coverage.

Figure 2 shows the numerical results comparing the downlink and uplink SINRs with respect to the variation of *d*. The result is based on (10) and (13). To investigate the effect of *d* to SINR, *α* and *β* are fixed as 0.01. Other parameters are also fixed, as defined in Table 2. As shown in Figure 2, the downlink and uplink SINRs are the same when *d* is 2*r*_*e*_. However, the more *d* becomes lower than 2*r*_*e*_, the better the uplink SINR becomes than the downlink SINR, and vice versa. For example, the uplink SINR is higher than the downlink SINR by 6 dB when *d* is 1.5*r*_*e*_. The downlink SINR is also higher than the uplink SINR by 6 dB when *d* is 3*r*_*e*_. Note that the uplink SINR decreases more rapidly in inverse proportion to *d* than does the downlink SINR. As defined in (9), the downlink interference is affected only by *d*. The downlink SINR decreases when *d* increases, and consequently, the transmission power of the neighbor BSs also increases. However, the uplink SINR is affected by two different factors: the transmission power of the MSs in neighbor cells and the interference region, as defined in (11) and (12). When *d* increases, the transmission power of the MSs in neighbor cells and in the interference region increase, and consequently, the uplink SINR at a serving BS decreases more rapidly. For these reasons, the downlink and uplink asymmetric channels can be created by their different distances among the BSs.

**Figure 3:**
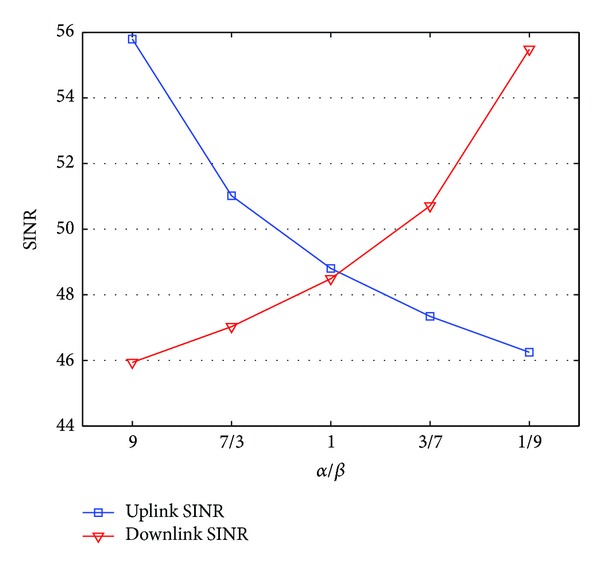
SINR according to uplink traffic load.

Figure 3 shows the numerical results for the downlink and uplink SINRs with respect to the ratio of downlink and uplink traffic loads. To examine the effects of *α* and *β* on SINR, *d* is fixed to $\sqrt{3}$ km. The other parameters have the same values as shown in Table 2, except *α* and *β*. As shown in Figure 3, the uplink and downlink SINR models are inversely proportional to *α* and *β*, respectively. The mean uplink SINR is higher than the mean downlink SINR by 10 dB when *α* is 0.0009 and *β* is 0.001. Similarly, the mean uplink SINR is lower than the mean downlink SINR by 9 dB in the case that *α* is 0.0001 and *β* is 0.009. As specified in (9) and (12), if *α* becomes larger than *β*, then the downlink interference power increases more rapidly than the uplink interference power, and vice versa. The reason is that the MS in neighbor cells also uses more carriers to communicate with the BS in neighbor cells, and the probability that the desired MS in the center cell uses the same frequency as the MSs in neighbor cells increases. Thus, an asymmetric channel can frequently be formed by *α* and *β* with different values according to the application types. Especially, the most popular services in the latest cellular systems, such as social network service (SNS), user created contents (UCC), or personal broadcasting service, generate asymmetric traffic in the downlink (*α*) and the uplink (*β*). Consequently, these services can create asymmetric channel conditions.

## 3. Proposed Handoff Scheme

### 3.1. Basic Idea

**Figure 4:**
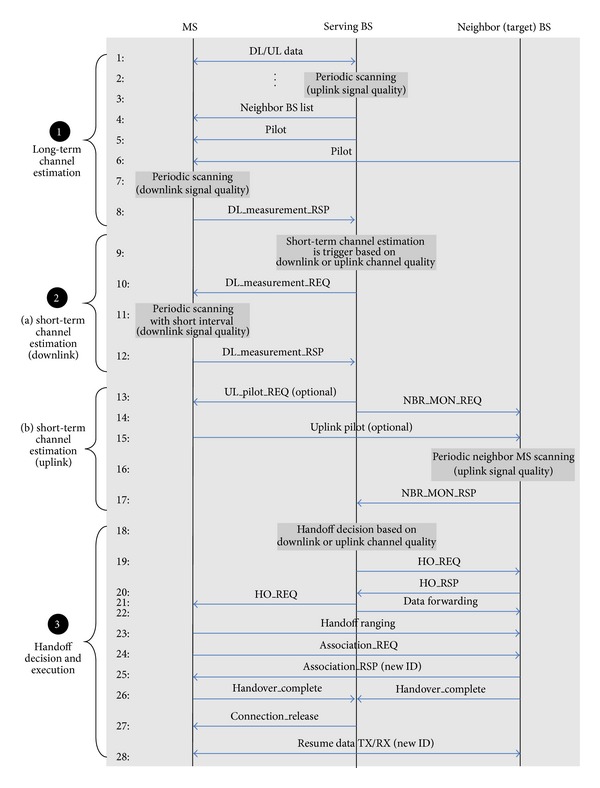
The proposed handoff procedures and signaling.

The basic idea of the proposed handoff scheme is to exploit uplink signal quality in addition to downlink signal quality in handoff decisions to cope effectively with asymmetry in downlink-uplink channels. An active state mobile station, connected to the uplink and downlink channels, is the main focus of the proposed scheme.  Figure 4 illustrates the handoff procedures and signaling of the proposed handoff scheme. As shown in the figure, the handoff procedures can be divided into three steps: long-term channel estimation, short-term channel estimation, and handoff decision and execution. In the conventional mobile-assisted handoff, an MS estimates only the downlink channel quality during the channel estimation step. However, in the proposed scheme, the serving BS periodically estimates the uplink channel quality from the uplink traffic channels of an active MS, and the MS periodically scans the downlink channel quality from the pilot channels of a serving BS and neighbor BSs. Based on the long-term channel estimation result, a short-term estimation process is triggered by the serving BS. In the conventional handoff scheme, a short-term estimation process and handoff decision are triggered only when the downlink channel quality is worse than a threshold. However, a short-term estimation process is triggered by the degradation of the uplink channel quality, as well as the downlink channel quality, in the proposed handoff scheme as illustrated in step 1 of Figure 4. The proposed scheme also determines the timing and direction of handoff based on the downlink and uplink channel qualities, in the short-term estimation process. Once the handoff execution time and direction are determined, the serving BS checks whether a target BS can accept a handoff call. Only when the target BS can accept the handoff call, the serving BS directs handoff execution to the MS. After receiving the handoff direction from the serving BS, the MS performs handoff ranging and association with the target BS. The uplink and downlink data transmission for the MS is suspended during the handoff execution step and resumed after handoff completion. The specific processes of each handoff step are given in the following sections.

### 3.2. Channel Quality Estimation

Channel quality estimation is one of the most important processes to affect the handoff performance. Normally, channel estimation comprises long-term and short-term estimations. Every mobile station in an active state estimates downlink channel quality over a relatively long-term period. The typical channel estimation period is 0.32, 0.64, 1.28, or 2.56 sec for a serving cell and 1.28 or 2.56 sec for neighbor cells [17]. Based on the long-term estimation result, a mobile station with channel quality worse than a predetermined threshold performs short-term estimation. In short-term estimation, the mobile station scans the channel quality more frequently. A time-averaged hysteresis value of short-term channel estimation is mainly used to determine a handoff execution. This section describes the downlink and uplink channel estimation methods in detail.

In Figure 4, step 1 illustrates the long-term channel estimation processes. As depicted in the figure, the proposed scheme defines both downlink and uplink channel estimation processes, while the conventional mobile-assisted handoff scheme defines only a downlink channel estimation process. In the conventional handoff schemes, only downlink channel quality is used to determine a handoff. However, the proposed handoff scheme exploits uplink channel quality as well and so defines the uplink channel estimation process. The downlink channel estimation process of the proposed scheme is the same as the conventional scheme. In the long-term estimation step, the serving BS broadcasts a list of neighbor BSs whose downlink channel quality should be scanned. An MS periodically scans downlink pilot channels from the serving BS and neighbor BSs and estimates their current downlink channel quality. The uplink channel estimation process, which is newly required in the proposed scheme, is performed by the serving BS. In the long-term estimation step, the serving BS periodically estimates the uplink channel quality of the MS that is in an active state by scanning the uplink traffic channel. As previously described, the primary focus of the proposed scheme is the MSs in an active state. Thus, uplink channel estimation is made possible without additional overhead, by using the uplink channel traffic. The long-term channel estimation result determines whether short-term channel estimation is required. The proposed handoff scheme triggers short-term channel estimation when the downlink or uplink signal quality becomes worse than a predetermined threshold. The condition to start a short-term channel estimation process is as follow:

(14) $$\begin{array}{c} \text{D}{\text{Q}}_{\text{serving}}<T_{1}\;\text{or}\;\text{U}{\text{Q}}_{\text{serving}}<T_{2}, \end{array}$$

where DQ_serving_ and UQ_serving_ are the downlink and uplink channel qualities, respectively, with a serving BS. Note that DQ_serving_ and UQ_serving_ are time-averaged hysteresis values for a given period in order to avoid a ping-pong effect [18] and frequent handoff. The thresholds, *T*_1_ and *T*_2_, are determined by the QoS requirement of each service type. For example, if the minimum downlink SINR for a certain data service is 5 dB, then *T*_1_ should be sufficiently larger than 5 dB.

Once the short-term channel estimation process is triggered, the MS or the serving BS should monitor the downlink and uplink channel qualities more frequently, as depicted in step 2 of Figure 4. The short-term downlink channel estimation process is the same as in the long-term case, except for the estimation cycle. However, the uplink channel estimation process is quite different from the long-term case. In short-term estimation, the serving BS requests the neighbor BSs to estimate the uplink channel quality of the MS, as depicted in step 2(b) of Figure 4. Unlike the serving BS, the neighbor BSs require an additional mechanism to measure the uplink signal quality of the MS because there is no connected channel between the MS and the neighbor BS. The simplest way is the serving BS-assisted method. If the serving BS informs the neighbor BSs for the MS identification and uplink channel allocation, then the neighbor BSs can scan the designated uplink channel quality of the MS. In this case, the accuracy of the channel estimation is limited because the MS might not use the entire frequency band to transmit data, and other MSs in the neighbor BSs might use the same uplink channel at the same time. Therefore, to increase the uplink channel estimation accuracy, an additional scanning mechanism or extra uplink pilot channel is required. Similar to an uplink channel sounding described in IEEE 802.16e [13], the MS can transmit a pilot signal with a predetermined pilot pattern on the common uplink pilot channel that is shared among two or more adjacent BSs. Before an uplink pilot transmission, the serving BS informs the neighbor BSs about the user information and pilot pattern. Then the neighbor BSs can clearly detect the pilot signal and accurately estimate the uplink channel quality of the MS. Using the uplink pilot channel is more favorable for accurate channel estimation. However, it creates uplink overhead in the case of a large number of handoff users. To reduce the overhead, the serving and neighbor BSs can use common traffic channels instead of the pilot channel. The common traffic channels are allocated to the handoff users located in different cells in an orthogonal manner. The scheduling information of these channels is shared among the adjacent BSs. Then the neighbor BSs can precisely estimate the uplink channel quality of handoff users with reduced overhead. Based on the short-term channel estimation results, the timing and direction of the handoff is determined.

### 3.3. Handoff Decision and Execution

**Figure 5:**
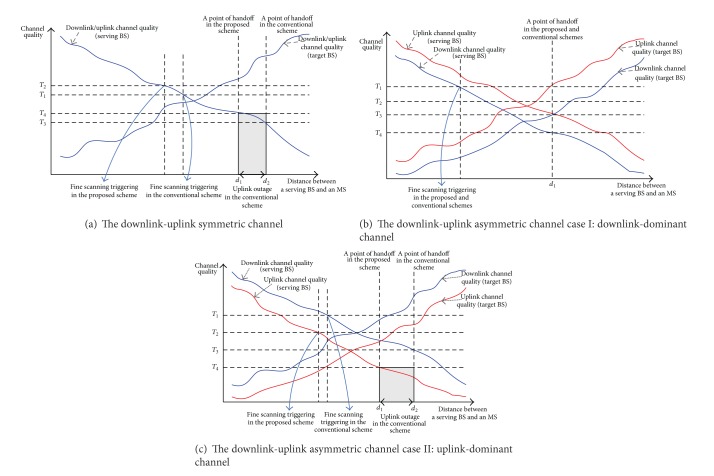
Handoff according to the downlink and uplink channel variations.

In the proposed handoff scheme, a handoff is triggered when the uplink or downlink channel quality is worse than a threshold for a certain period.  Figure 5 illustrates a point of handoff execution according to the downlink and uplink channel variations. Figures 5(a), 5(b), and 5(c) show a handoff triggering under a downlink-uplink symmetric channel, a downlink-dominated channel, and an uplink-dominated channel, respectively. A downlink-dominated channel means that the downlink channel quality is worse than a threshold, but the uplink channel quality is better than a threshold, and vice versa. For these channel types, the proposed handoff scheme defines different handoff decision criteria to determine an appropriate point of handoff execution. First, for the downlink-uplink symmetric channel, a handoff is executed when the following condition is satisfied after the short-term channel estimation:

(15) $$\begin{array}{c} \text{D}{\text{Q}}_{\text{serving}}<T_{3},\\ \left(\text{D}{\text{Q}}_{\text{target}}>T_{3\;},\;\;\text{U}{\text{Q}}_{\text{target}}>T_{4}\right)\;\left(\text{if}\;T_{3}\geq T_{4}\right)\;\;\text{or}\\ \text{U}{\text{Q}}_{\text{serving}}<T_{4},\\ \left(\text{U}{\text{Q}}_{\text{target}}>T_{4},\;\;\text{D}{\text{Q}}_{\text{target}}>T_{3}\right)\;\left(\text{if}\;T_{3}<T_{4}\right), \end{array}$$

where *T*_3_ and *T*_4_ are the minimum downlink and uplink channel qualities to guarantee the required QoS, respectively. If the uplink or downlink channel quality is worse than the threshold for a given period, then a handoff-call is dropped. Thus, the MS should perform a handoff before the call is forcibly dropped. If the downlink QoS requirement is stricter than the uplink QoS requirement, then the downlink channel quality becomes a dominant handoff criterion, and vice versa. Note that the proposed handoff decision criterion is quite different from the conventional handoff scheme, even for the downlink-uplink symmetric channel because it takes into consideration the uplink QoS requirement to determine a handoff execution.  Figure 5(a) compares the handoff execution point between the proposed and the conventional handoff schemes under the downlink-uplink symmetric channel. In this figure, *T*_4_ is larger than *T*_3_; that is, the uplink QoS requirement is assumed to be stricter than the downlink QoS requirement. A handoff is triggered at *d*_1_ in the proposed scheme, whereas the handoff is triggered at *d*_2_ in the conventional scheme. The reason is that the proposed scheme uses the uplink QoS requirement *T*_4_ as a handoff criterion, but the conventional scheme uses the downlink QoS requirement *T*_3_ as a handoff criterion. As illustrated in Figure 5(a), a call drop can occur at *d*_1_ in the conventional handoff scheme, whereas the call is successfully sustained on a target cell in the proposed scheme.

The next interesting case is the downlink-dominated channel in which the downlink channel quality is worse than a predetermined threshold but the uplink channel quality is better than a predetermined threshold, as illustrated in Figure 5(b). In this case, the downlink channel quality is a dominant factor in determining a handoff execution, and a handoff takes place when the following condition is satisfied:

(16) $$\begin{array}{c} \text{D}{\text{Q}}_{\text{serving}}<T_{3},\;\;\left(\text{D}{\text{Q}}_{\text{target}}>T_{3\;},\;\;\text{U}{\text{Q}}_{\text{target}}>T_{4}\right). \end{array}$$

Figure 5(b) shows the handoff execution point with respect to the channel quality under the downlink-dominated channel. In Figure 5(b), the overall uplink channel quality is better than the downlink channel quality. However, the minimum requirement of the downlink channel quality is higher than the uplinks. In the downlink-dominated channel, the proposed scheme chooses the downlink channel quality as a handoff criterion, as in the conventional scheme. Therefore, the short-term channel estimation and handoff execution point are exactly the same as with the conventional handoff scheme. Note that a call drop can still occur in the conventional handoff scheme, even on the downlink-dominant channel, if the uplink QoS requirement is much stricter than the downlink QoS requirement.

Finally, under the uplink-dominated channel, only the uplink channel quality is worse than a predetermined threshold that guarantees the required QoS. Thus, the uplink channel quality is the handoff criterion in this case, and a handoff occurs when the following condition is satisfied:

(17) $$\begin{array}{c} \text{U}{\text{Q}}_{\text{serving}}<T_{4},\;\;\left(\text{U}{\text{Q}}_{\text{target}}>T_{4\;},\text{D}{\text{Q}}_{\text{target}}>T_{3}\right). \end{array}$$

Figure 5(c) illustrates a handoff execution point for the uplink-dominated channel when the overall downlink channel quality is better than the uplink channel quality. In the conventional handoff scheme, the uplink channel quality has already been worse than the required threshold at *d*_1_ but the handoff is executed at *d*_2_, where the downlink channel quality becomes worse than the threshold *T*_3_. Thus, the call is forcibly dropped because of the poor uplink channel quality. However, the proposed handoff scheme can prevent a call drop by considering both downlink and uplink channel qualities in the handoff decision process to find an appropriate handoff execution point.

## 4. Performance Evaluation

We compare the performance of the proposed handoff scheme to the mobile assisted handoff (MAHO) that has been widely used in the contemporary cellular systems such as IEEE 802.16m/WiMAX [14, 19]. We built a computer simulator using OPNET for the performance evaluation.  Table 3 represents the simulation parameters [19–22]. In the simulation, 3-tier cell structure is modeled with 37 cells, taking into consideration the inter-cell interference. The MSs, which are traced to estimate the performance, move around and hand off within the first tier cells including a center cell. Dummy MSs are randomly distributed in the second and third tier cells for uplink interference. Inter-cell interferences are arisen from the adjacent cells and the neighbor cells after next. To mitigate any ping-pong effect, we calculate the moving average of the measured SINR and use the averaged SINR for handoff initiation and decision thresholds. In the simulation, the averaging window is 500 msec that is one of the recommended value in 3GPP-LTE [17, 20]. Fast fading is not considered because it is lessened in the averaged SINR.

We investigate the handoff-call dropping probability and the service interruption time during handoff as performance metrics. A tolerable service interruption time is typically less than 150 msec for seamless service [19]. Thus, it is assumed that a handoff-call dropping arises when the downlink or uplink signal level is lower than the dropping threshold during consecutive 30 frames. A handoff-call dropping probability is the ratio of the number of handoff outages to the number of handoff trials. The service interruption time is defined as the duration from an *HO_REQ* message to *Resume_data_TX/RX* in the handoff procedures shown in Figure 4.

**Figure 6:**
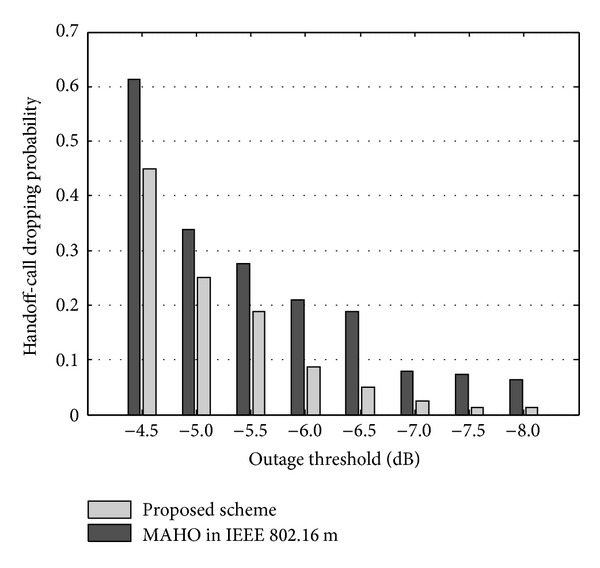
Handoff-call dropping probability with respect to dropping threshold (case 1: low-density scenario).

Handoff-call dropping probability is evaluated for two scenarios in which MSs are distributed with low-density and high-density on cell-edge area. In the simulation, number of MSs per cell is assumed to be 10 for two cases. In low-density case, MSs are uniformly distributed on overall cells. However, in high-density case, MSs are distributed only on cell-edge area that is region *e* of Figure 1. *T*_3_ and *T*_4_ are assumed to be −6.5 dB which is the lowest adaptive modulation and coding (AMC) level defined in [17, 20].  Figure 6 compares the handoff-call dropping probabilities of the proposed and conventional mobile-assisted handoff (MAHO) in IEEE 802.16m in case of the low-density scenario. In the simulation results, the proposed scheme shows a lower handoff-call dropping probability compared to MAHO in IEEE 802.16m. This result chiefly centers on the fact that the conventional MAHO cannot prevent a handoff-call dropping for uplink-downlink channels asymmetry when the downlink channel quality is acceptable while the uplink channel quality is worse than the threshold. However, the proposed scheme copes with both uplink and downlink signal degradations and consequently reduces the handoff-call dropping under an uplink-dominant channel.

**Figure 7:**
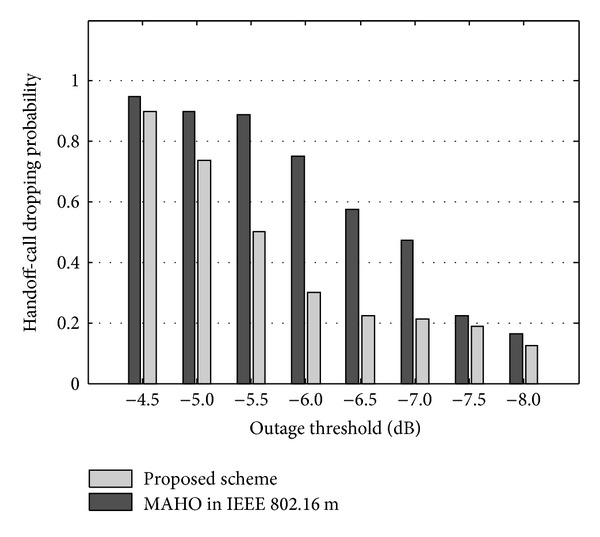
Handoff-call dropping probability with respect to dropping threshold (case 2: high-density scenario).

Figure 7 shows the simulation results for the high-density scenario. The uplink interference from the users on neighbor cells increases proportionally to the population on the cell-edge area. Thus, the inter-cell interference of the uplink is increased in the high-density scenario, and consequently, the channel asymmetric condition occurs more frequently. As a result, the proposed scheme shows much better performance than the conventional MAHO in the high-density scenario. This result indicates that the proposed scheme would be more effective in densely populated urban areas.

**Figure 8:**
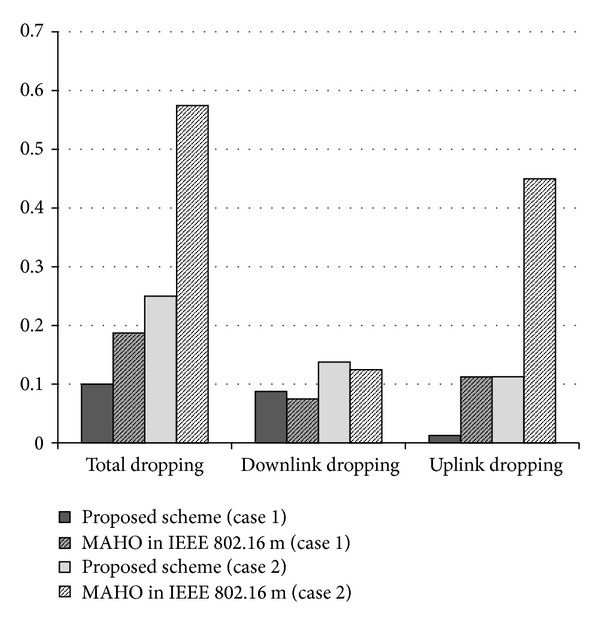
Handoff-call dropping probability caused by uplink signal dropping or downlink signal dropping.

Figure 8 shows the handoff-call dropping probability on uplink or downlink channels in the handoff region. In this simulation, the uplink and downlink dropping threshold is fixed as −6.5 dB. In the low-density scenario (case 1), the downlink dropping probability is almost the same in the proposed as in the conventional scheme. However, the proposed scheme decreases the uplink dropping probability by 10% compared to the conventional scheme and therefore reduces the total dropping probability by 9%. Exploiting uplink channel quality during the handoff decision process can decrease the uplink dropping under the uplink-dominant channel. In the high-density scenario (case 2), the downlink dropping probability shows almost the same pattern as the low-density scenario. However, the proposed scheme significantly reduces the uplink dropping probability in the high-density scenario: by 34%, compared to the conventional scheme. As described in the previous section, uplink inter-cell interference is increased in high-density cells, making uplink-dominant channels more likely. Thus, the proposed scheme is more effective under uplink-dominant asymmetric channels, and it reduces the total handoff-call dropping probability by 33%, compared to the conventional scheme. This result demonstrates that the proposed scheme is feasible and effective for uplink-downlink asymmetric channels, especially for the uplink-dominant channel or uplink-dominant services.

**Figure 9:**
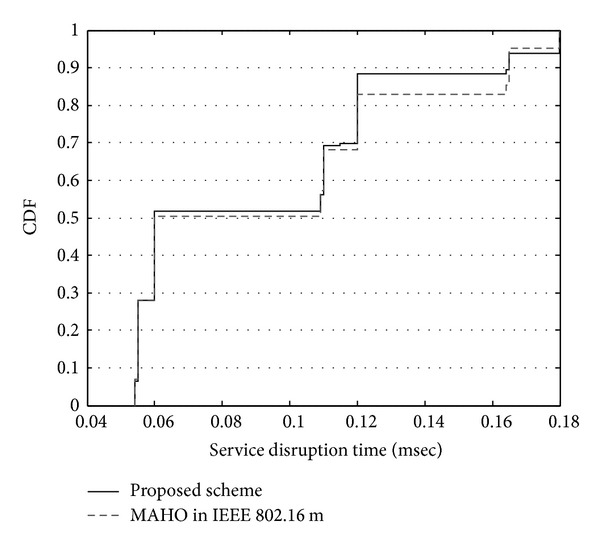
Service interruption time.

Figure 9 shows the cumulative density function of the service interruption time caused by handoff. In the conventional scheme, 83% of users satisfy the 150 msec requirement, which is the typical requirement of the service interruption time during a handoff process. The other 17% of users experience service interruption time of more than 180 msec. This is caused by the loss of handoff ranging messages that are transmitted by a handoff user to a target BS. Handoff ranging messages can be dropped due to an inaccurate adjustment of uplink transmission power. The conventional handoff scheme adjusts the handoff ranging power based on the downlink channel quality. In contrast, with the proposed scheme, 89% of users experience less than 150 msec of service interruption time because the proposed scheme adjusts the handoff ranging power according to the uplink channel quality as well as the downlink channel quality. In the simulation, the service interruption times of handoff users who retransmit a handoff ranging message once or twice are about 120 msec and 180 msec, respectively.

## 5. Conclusions

This paper proposes an enhanced handoff scheme for the downlink-uplink asymmetric channel in the latest cellular systems. We first derive the mathematical models of the downlink and uplink SINRs to demonstrate the occurrence of asymmetric channels. The numerical results show that an asymmetric channel can frequently occur with asymmetric traffic services like SNS and UCC. An asymmetric channel can also be created by their different distances among the BSs. However, most conventional handoff schemes have been designed on the assumption that the uplink channel quality is usually servile to the downlink channel quality, and accordingly, the handoff schemes are based solely on the downlink channel quality. Therefore, the handoff-call dropping probability may be higher under asymmetric channel conditions, compared to symmetric channel conditions, when using the conventional handoff schemes. To overcome these problems, an enhanced handoff scheme is proposed to exploit the uplink channel quality in addition to the downlink channel quality in determining appropriate handoff timing and direction. The simulation results show that the proposed handoff scheme reduces the handoff-call dropping probability and the service interruption time caused by handoff on asymmetric channels. The latest wireless data services, including SNS, wireless P2P, and personal broadcasting services, can improve their performance with use of the proposed scheme. Moreover, the proposed scheme can be easily extended for Wi-Fi systems.

## Figures and Tables

**Table 1:** Notations for system model.

Notation	Description
*d*	Distance between the BS in the center cell and the BS in a neighbor cell
*b*	Index of the BS in the center cell
*m*	Index of the MS in the center cell
*n*	Index of the MS in the neighbor cell
*z*	Index of the BS in the neighbor cell
*r*_*h*_	Distance between the center and the vertex of a hexagonal cell
*r*_*e*_	Radius of an equivalent circle to a hexagonal cell
*R*_1_	Distance between a BS and an MS in the center cell
*R*_2_	Distance between a BS in a neighbor cell and an MS in the center cell
*R*_3_	Distance between a BS in the center cell and an MS in a neighbor cell
*γ*	Cell Propagation loss factor (=−4)
*ε*	Minimum distance between a BS and an MS
*P*_max,BS_serving__^(*t*)^	Maximum transmission power of a BS in the center cell
*P*_max,MS_serving__^(*t*)^	Maximum transmission power of an MS in the center cell
*P*_max,BS_neighbor__^(*t*)^	Maximum transmission power of a BS in a neighbor cell
*P*_max,MS_neighbor__^(*t*)^	Maximum transmission power of an MS in a neighbor cell
*ζ*_*b*→*m*_	Shadow fading from BS b to MS m
*θ*	Main lobe width of sectored cells
$\overset{-}{\delta }$	Average number of active MSs in a cell
*α*	Downlink resource allocation ratio per MS
*β*	Uplink resource allocation ratio per MS

**Table 2:** Simulation parameters.

Parameter	Value
*α*	0.01
*β*	0.01
*θ*	120°
*γ*	−4
*N*_0_	−174 dBm
*P*_max,BS_^(*t*)^	39, 42, 48 dBm
*P*_max,MS_^(*t*)^	27, 30, 36 dBm
*σ*_*ζ*_	8 dB
*ε*	10 m
$\overset{-}{\delta }$	100

**Table 3:** Simulation environment and applied parameters.

Simulation parameters	
Number of BSs	7 active BSs, 30 dummy BSs
Number of MSs	10 MSs per a cell
Cell coverage	1 km^2^
Velocity	60 km/h (Uniform dist.)
Path loss exponent	4
Shadowing model	Log-normal distance (mean: 0, standard deviation: 8 dB), correlation distance: 50 m
Thermal noise density	−174 dBm/Hz
Moving average window size	100 frames

System parameters	

Bandwidth	10 MHz
Frame size	5 msec
Processing time	10 msec
Transmission delay	5 msec
Decision hysteresis	3 dB
Synchronization	5 msec
RF switching time	5 msec
